# Adverse pregnancy outcomes in women with diabetes

**DOI:** 10.1186/1758-5996-4-41

**Published:** 2012-09-11

**Authors:** Carlos Antonio Negrato, Rosiane Mattar, Marilia B Gomes

**Affiliations:** 1 Bauru's Diabetics Association, Department of Internal Medicine, Rua Saint Martin 27-07 CEP 17.012-433, Bauru, São Paulo, Brazil; 2 Department of Gynecology and Obstetrics, São Paulo Federal University, São Paulo, Brazil; 3 Department of Internal Medicine, Diabetes Unit, State University Hospital of Rio de Janeiro, Rio de Janeiro, Brazil

**Keywords:** Type 1 diabetes, Type 2 diabetes, Gestational diabetes, Diabetic pregnancy, Pregnancy adverse outomes

## Abstract

Pregnancy affects both the maternal and fetal metabolism and even in nondiabetic women exerts a diabetogenic effect. Among pregnant women, 2 to 17.8% develop gestational diabetes. Pregnancy can also occur in women with preexisting diabetes, that can predispose the fetus to many alterations in organogenesis, growth restriction and the mother to some diabetes-related complications like retinopathy and nephropathy or accelerate the course of these complications if they are already present. Women with gestational diabetes generally start their treatment with diet and lifestyle modification; when these changes fail in keeping an optimal glycemic control, then insulin therapy must be considered. Women with type 2 diabetes in use of oral hypoglycemic agents are advised to change to insulin therapy. Those with preexisting type 1 diabetes must start an intensive glycemic control, preferably before conception. All these procedures are performed aiming to keep glycemic levels normal or near-normal as possible to avoid the occurrence of adverse perinatal outcomes to the mother and to the fetus. The aim of this review is to reinforce the need to improve the knowledge on reproductive health of women with diabetes during gestation and to understand what are the reasons for them failing to attend for prepregnancy care programs, and to understand the underlying mechanisms of adverse fetal and maternal outcomes, which in turn may lead to strategies for its prevention.

## Background

Pregnancy affects both the maternal and fetal metabolism and even in nondiabetic women exerts a diabetogenic effect. As normal pregnancy progresses insulin resistance increases and pancreatic β-cells reserve is stressed aiming to maintain glycemia within normal ranges; gestational diabetes results when β-cells fail to maintain glycemia in these ranges. At delivery, when the placenta that exerts the major anti-insulin effect is removed, usually glucose homeostasis is restored. However, 2 to 17.8% of women develop gestational diabetes, depending on the diagnostic criteria used and the studied population; gestational diabetes represents a very strong predictor for the development of permanent diabetes later in life [1].

Besides gestational diabetes, pregnancy can also occur in women with preexisting diabetes. A significant increase in preexisting diabetes during pregnancy has been observed in the USA between 1999 and 2005, rising from 10% to 21% [2]. Pregestational diabetes, both type 1 and type 2 can cause alterations from fertilization, through all pregnancy period and even after its end. It can predispose the fetus to many alterations in organogenesis, growth restriction and predispose the mother to some diabetes-related complications like retinopathy and nephropathy or accelerate the course of these complications if they are already present. Gestational diabetes generally leads to fetal growth alterations [1].

The aim of this review is to report the literature on the great incidence of maternal and fetal adverse pregnancy outcomes found nowadays. All these adverse outcomes are closely related to poor glycemic control during the organogenesis period and unfortunately are still very prevalent. Hyperglycemia during the periconceptional period is probably the major teratogenic existing factor, but obesity, hypertension and other factors associated with the metabolic syndrome might also be relevant [1].

### Fetal and maternal adverse outcomes in pregnancies with diabetes

Normal pregnancy is a non pathological condition characterized by a series of complex hormonal adaptations that occur to ensure that sufficient glucose is available to meet the nutritional requirements of the growing fetus without causing maternal hypoglycemia. Hyperglycemia, a hallmark of diabetes, is an important cause of maternal and fetal complications in pregnancies of women with any type of diabetes. Generally, the pregnancy in women with diabetes is associated with increased risk of obstetric and neonatal complications, morbidity and mortality [1-19].

The most common fetal adverse outcomes found in pregnancies of women with diabetes are fetal and neonatal loss, a great variety of congenital abnormalities and malformations, premature delivery (delivery occurring before 37 weeks’ gestation), fetal growth acceleration and macrosomia (defined as a birth weight above 4 kg and/or >90th percentile weight for gestational age or large for gestational age) which are associated with several obstetric complications like birth trauma, hypertrophic miocardiopathy, stillbirth, respiratory distress syndrome, neonatal hypoglycemia, hypocalcemia, hyperbilirubinemia and polycythemia; maternal complications are pregnancy induced hypertension, pre-eclampsia, hemolysis, elevated liver enzymes, low platelets (HELLP) syndrome, Cesarean section, hypoglycemia and the worsening of any degree of a pre-existing renal insufficiency and retinopathy [20,21].

### Fetal adverse outcomes

Pregnancy loss is significantly higher among women with diabetes compared to the nondiabetic population [14]. Recently, a population-based cohort study conducted in the UK by Casson et al. has shown that women with type 1 diabetes have a higher risk of late fetal loss, presenting a four- to five-fold increase in perinatal death, and a four- to six-fold in stillbirth [8] compared to the general population. Neonatal mortality is also higher among infants of diabetic mothers in approximately 15-fold when compared to the general population [14].

Schaefer et al. have found a two-fold increase in the risk of congenital anomalies when fasting glucose levels are already greater than 120 mg/dL when first detected during pregnancy [22]. The increased risk of congenital abnormalities found in diabetic mothers seems to be associated to poor metabolic control during the period of organogenesis that occurs in the first trimester of pregnancy probably due to the negative impact of a hyperglycemic milieu in the growing fetus [23].

The pathogenesis of congenital malformations of all types, which have four to ten times higher incidence in pregnant women with diabetes, is very complex and has possibly a multifactorial origin [8,14,23]. A strong link between hyperglycemia and malformations has been established, but the precise mechanism by which it occurs has not been completely elucidated. It is supposed that hyperglycemia could cause damage to the developing yolk sac, an increased production and liberation of free oxygen radicals, deficiency of myoinositol and arachidonic acid and a disruption in signal transduction; increasing evidences suggest that embriopathies might be connected to a disruption in intracellular signaling by inositol-derived effectors and prostaglandin precursors such as arachidonic acid. Also, a as a result of the presence of these fuels, some type of genotoxic effect might occur which could cause morphologic damages in the fetus [23-27].

Nowadays, there is compelling evidence linking epigenetic factors to many human diseases including diabetes. Epigenetic factors, by different types of reactions, could mediate the interplay between genes and environment resulting in activation or repression of genetic transcription, or even silencing the genetic transcription. The most important epigenetic reactions affecting genetic transcription are acetylation and methylation. These reactions occur mainly in the tail of histones that are proteins where DNA is wrapped. Brownlee et al. [28] have demonstrated in human aortic endothelial cells, that excessive concentration of reactive oxygen species (ROS) resulting from hyperglycemia can induce monomethylation of lysine from histone 3 increasing the expression of the subunit p65 of nuclear factor kappa beta. This reaction is responsible for the increased transcription of vascular cell adhesion molecule 1 and monocyte chemoattractant molecule 1 (MCP1) that are both related to hyperglycemia-induced arterial pathology [28]. However, this reaction persisted after a six days period of subsequent normoglycemia, supporting the concept of metabolic memory. Another study showed an increase of DNA methylation in insulin promoter DNA in human pancreatic islets which was negatively correlated with insulin gene expression and positively correlated with HbA1c levels [29].

Specifically concerning GDM, some epigenetic alterations have been described recently mainly related to beta cell function and intrauterine growth retardation. These alterations could result in reduction of expression of PDX-1, a transcription factor that regulates beta cell development [30]. Although further studies are necessary to establish the possible role of PDX-1 in GDM, it is important to note that epigenetic effects are defined as heritable changes to DNA structure that do not involve changes to the DNA sequence; being so, they can be reset or undone under certain conditions such as in early development [30]. Previous studies showed that folic acid, that is a methyl donor, prevents genomic damage in human lymphocytes in vitro and maybe also the genotoxicity, cytotoxicity, and might have cytostatic effects on the human genome [31].

Recently, the effect of folic acid on human reproduction has been well reviewed and the relationship between folate with vitamin B12 upon DNA synthesis and methylation has been demosntrated [32]. Randomized trials have shown that periconceptional supplementation with folic acid can reduce the frequency of midline embryonic defects, as well as heart defects, orofacial clefts and miscarriages [32,33]. During pregnancy, folic acid requirements increase due to expansion of maternal erythrocyte number, uterine enlargement, placental development and fetal growth [33]. Folic acid deficiency during gestation is associated with impaired cellular growth and replication that can result in megaloblastic anemia, spontaneous abortions, fetal malformations, placental abruption, preterm delivery and low birth weight [32]. Many Medical Societies like the American College of Obstetricians and Gynecologists [34] and Canadian Obstetrical Societies [35] recommend high-dose folic acid supplementation (4–5 mg/day) in diabetic women before and during pregnancy aiming to reduce the risk of congenital malformations in their babies; this dose is ten times higher than those recommended for non diabetic women [34,35].

The major congenital malformations among offspring of women with diabetes are found in the following systems: cardiovascular (cardiac transposition of the great arteries, ventricular septal defect, coarctation of the aorta, atrial septal defect, asymmetric septal hypertrophy), central nervous system (neural tube defects, including anencephaly, microcephaly and isolated hydrocephalus), gastrointestinal (duodenal atresia, anorectal atresia, hypoplastic left colon), musculoskeletal (talipes, arthrogryposis), urinary tract (uretal duplication, cystic kidney, renal dysgenesis, hydronephrosis), caudal regression syndrome, cleft lips and palate anomalies [9,20]. These major congenital abnormalities are an important contributory factor for the high mortality rates found in infants of women with diabetes [8]. Preterm delivery is four to five times higher among mothers with diabetes [3]. Generally, babies of mothers with diabetes tend to fare less well at all stages of the pregnancy. The possibility of a premature delivery is always strived by the health care team as much as possible, but this is not always possible. In the presence of significant complications, the mother's and the babies' health is paramount and early delivery may become a life-saving measure [3]. Preterm infants born to women with any type of diabetes, have a much greater risk of presenting a wide range of complications such as, intrauterine growth restriction, low-birth-weight, respiratory distress syndrome, hypoglycemia, hypocalcemia, polycythemia, intrauterine death, hyperbilirubinemia, several types of malformations, hypertrophic cardiomyopathy and asphyxia compared to those born to women without diabetes [1,3].

Glucose is transported freely across the placenta by facilitated diffusion; in the presence of maternal hyperglycemia large amounts of glucose reach the fetus which leads to fetal hyperinsulinemia that causes fetal overgrowth and/or macrosomia. Increased glucose levels, even those below the threshold for gestational diabetes, for example, are associated with continuous increase in the risk of macrosomia and Cesarean section as described by Sermer et al. [36] and by the Hyperglycemia and Adverse Pregnancy Outcomes (HAPO) Study [37]. Any degree of hyperglycemia that the fetal tissues are exposed to, stimulates insulin secretion that can cause hypertrophy of insulin-sensitive tissues such as adipose tissue, skeletal and myocardial muscle, hepatic tissues and even islets of Langherhans in the pancreas; as a result, an accelerated fetal growth can be found and eventually macrosomia. Besides high blood glucose levels, high levels of other metabolic fuels such as some aminoacids and free fatty acids might as well increase fetal insulin secretion after beta cell replication [38,39].

The great majority of macrosomias (70%) are due to constitutional and genetic factors, prolonged pregnancies or to some syndromes [40]. Macrosomias occurring in children of mothers with diabetes correspond to 30% of the cases; these macrosomias are classically dysmorphic with a greater growth of shoulders and abdomen in relation to head [41], what increases the risk of several obstetric complications, such as higher rates of Cesarean section, shoulder dystocia, chorioamnionitis, severe perineal lacerations and postpartum hemorrhage [17,19]. The rates of macrosomia are 3.5–4.5 times greater among infants of women with pregestational diabetes than those found in infants born to nondiabetic mothers [17,19].

The newborn's heart has a great amount of insulin receptors. In a state of hyperinsulinism such as gestational diabetes, a hypertrophic miocardiopathy can occur; this condition is characterized by hypertrophy of the septum and ventricular walls that can in some cases obstruct blood flow. It can be asymptomatic, in some instances cause cardiac failure or regress in the first months of life. It is also thought to be an important cause of intrauterine death and stillbirth [42].

The excess of insulin in the fetal circulation can delay pulmonary maturation associated with the low production of surfactant leading to the respiratory distress syndrome or hyaline membrane disease. This condition is about six-fold more frequently found in newborns of women with diabetes than in nondiabetic women [43].

Hypoglycemia is the most common neonatal complication that occurs in diabetic pregnancies. Right after the section of the umbilical cord, the deprivation of maternal glucose supply, can lead to this condition that generally happens in the first hours of life. It can be asymptomatic or be accompanied by lethargy, agitation and even convulsions. It must be prevented and recognized early because it can have severe implications for the newborn’s immediate and future health [39,44].

Also after the section of the umbilical cord, the deprivation of maternal nutrient flow, can lead to hypocalcemia (Ca++ <7 mg/dL) and hypomagnesiemia (Mg++ <1.5 mg/dL); these conditions also generally happen in the first hours of life and can cause neuromuscular excitability, irritability, apnea and even convulsions [39,45,46].

Chronic hyperinsulinemia can lead to an increased erythropoiesis and also to an accelerated hemolysis due to glycation processes, that associated to prematurity, modified hepatic conjugation and modification of the entero-hepatic circulation, that are frequently found in newborns to mothers with diabetes, can lead to hyperbilirubinemia (bilirubin >13 mg/dL); this condition is two-fold more frequently found in newborns of mothers with diabetes compared to the background population [47].

Generally, the placental blood flow is impaired and transplacental exchanges are poor in the presence of hyperglycemia and hyperinsulinemia and a state of chronic relative hypoxemia occurs. Under the acute and chronic stimulus of hypoxia, extramedullary erythopoiesis is activated and a consequent polycythemia develops. It is diagnosed by a venous hematocrit > 65% or hemoglobin > 20 g/dL. This occurs in about 30% of newborns of mothers with diabetes [48].

The metabolic risks for the infants of mothers with diabetes extends much longer after birth; the influence of the maternal in utero environment is evidenced in the U- or J-shaped relationship between birth weight and adult obesity and metabolic disease demonstrating that both a limited or excessive nutritionally intrauterine environment can lead to postnatal obesity and related chronic diseases, like type 2 diabetes, hypertension and metabolic syndrome. This might result in a vicious cycle that could explain the great increase in the rates of obesity, gestational diabetes and type 2 diabetes seen in the last decades [38,49,50].

### Maternal adverse outcomes

On the maternal side, morbidity and mortality rates are also higher among pregnant women with diabetes. Rates of pre-eclampsia (12.7%), Cesarean section (44.3%) and maternal mortality (0.6%) found among women with type 1 diabetes are considerably higher than in the background population. Hypertension and postpartum hemorrhage are more likely to be found in pregnancies complicated by diabetes [1,3]. Pregnant women with type 1 diabetes present a death rate 109 times greater than the general population and 3.4 times greater than in nonpregnant type 1 diabetic women [18].

Changes in glucose disposal and insulin kinetics seen in pregnancy have special importance for women with pregestational diabetes because hypoglycemia, many times of severe intensity, can occur generally in early pregnancy, a period when insulin requirements may decrease, possibly because of nausea and vomiting, compared to prepregnancy and to the second half of pregnancy; it is a dangerous condition that can leave important sequellae to the mother and the fetus [51].

The prevalence of chronic hypertension, gestational hypertension, pre-eclampsia, and superimposed pre–eclampsia are all more frequent in diabetic pregnancies. The presence of chronic hypertension, microalbuminuria or diabetic nephropathy should be evaluated in early pregnancy. All pregnant diabetic women should receive strict metabolic control and be intensively monitored for the development of pre–eclampsia and pregnancy induced hypertension because these conditions can cause a negative impact for the mother’s and the fetus’ health [52].

Patients presenting moderate -to- severe renal insufficiency and uncontrolled hypertension prior to conception and/or early in pregnancy show an increased risk for accelerated progression to end-stage renal disease. The perinatal survival is high (> 95%) in patients with minimal renal dysfunction (serum creatinine < 125 μ mol/L [ < 1.4 mg/dL]); however, pre-eclampsia and preterm delivery are very common conditions found in this group of patients [53].

Diabetic retinopathy, a very prevalent microvascular complication of diabetes, remains the leading cause of acquired blindness in young and middle - aged adults in the world. Pregnancy, a condition that presents hormonal, hemodynamic, metabolic, and immunologic changes, is a risk factor for progression of diabetic retinopathy. The etiology of retinopathy acceleration during pregnancy is still unknown; proposed mechanisms involve rapid improvement in glycemic control, altered hemodynamic properties, with the reduction of blood flow in the retina and immuno-inflammatory processes [54].

## Conclusion

Pregnancy should be planned in women with preexisting diabetes, which includes a strict metabolic control with near or near-normal glucose levels, reached through lifestyle modifications, a healthy diet, and an exercise planning program with the supplementation of folic acid that is advocated to prevent malformations and miscarriages.

Future research is warranted in this field and two areas must be addressed. First, there are the social issues related to diabetes in pregnancy, in women with preexisting diabetes and in those with gestational diabetes. There is a need to increase knowledge on reproductive health of these women and to understand what are the reasons for them failing to attend for prepregnancy care, to change their lifestyle, to allow the development of more appropriate facilities and methods which should improve uptake and the final results. Another social issue is trying to address the obesity epidemic which is directly related to the occurrence of gestational diabetes and may increase the risk of malformations, and may also make their antenatal detection and diagnosis more difficult. The second area is for emphasizing the importance of basic research into the mechanisms causing adverse fetal and maternal outcomes, which in turn may lead to more efficient strategies for their prevention.

## Competing interests

The authors declare that they have no competing interests.

## Authors' contributions

All authors participated equally in the development of this paper. All authors also read and approved the final manuscript.
